# Inhibition of Necroptosis Rescues SAH-Induced Synaptic Impairments in Hippocampus via CREB-BDNF Pathway

**DOI:** 10.3389/fnins.2018.00990

**Published:** 2019-01-07

**Authors:** Chunlei Yang, Tong Li, Hao Xue, Lingxiao Wang, Lin Deng, Yunkai Xie, Xuemei Bai, Danqing Xin, Hongtao Yuan, Jie Qiu, Zhen Wang, Gang Li

**Affiliations:** ^1^Department of Neurosurgery, Qilu Hospital of Shandong University and Institute of Brain and Brain-Inspired Science, Shandong University, Jinan, China; ^2^Shandong Key Laboratory of Brain Function Remodeling, Jinan, China; ^3^Department of Neurosurgery, Qingdao Municipal Hospital, Qingdao, China; ^4^Department of Physiology, Shandong University School of Basic Medical Sciences, Jinan, China

**Keywords:** subarachnoid hemorrhage, early brain injury, necroptosis, hippocampus, synaptic impairment

## Abstract

Subarachnoid hemorrhage (SAH) is a devastating form of stroke that leads to incurable outcomes. Increasing evidence has proved that early brain injury (EBI) contributes mostly to unfavorable outcomes after SAH. A previously unknown mechanism of regulated cell death known as necroptosis has recently been reported. Necrostatin-1 (nec-1), a specific and potent inhibitor of necroptosis, can attenuate brain impairments after SAH. However, the effect of nec-1 on the hippocampus and its neuroprotective impact on synapses after SAH is not well understood. Our present study was designed to investigate the potential effects of nec-1 administration on synapses and its relevant signal pathway in EBI after SAH. Nec-1 was administrated in a rat model via intracerebroventricular injection after SAH. Neurobehavior scores and brain edema were detected at 24 h after SAH occurred. The expression of the receptor-interacting proteins 1 and 3 (RIP1and3) was examined as a marker of necroptosis. We used hematoxylin and eosin staining, Nissl staining, silver staining and terminal deoxynucleotidyl transferase dUTP nick end labeling (TUNEL) to observe the morphological changes in hippocampus. The protective effect of nec-1 on synapses was evaluated using western blotting and electron microscopy and Western blotting was used to detect the cAMP responsive element binding (CREB) protein and brain-derived neurotrophic factor (BDNF), and we used transmission electron microscopy and TUNEL to detect the protective effects of nec-1 when a specific inhibitor of CREB, known as 666-15, was used. Our results showed that in the SAH group, RIP1, and RIP3 significantly increased in the hippocampus. Additionally, injection of nec-1 alleviated brain edema and improved neurobehavior scores, compared with those in the SAH group. The damage to neurons was attenuated, and synaptic structure also improved in the Sham+nec-1 group. Furthermore, nec-1 treatment significantly enhanced the levels of phospho-CREB and BDNF compared with those in the SAH group. The protective effect of nec-1 could hindered by 666-15. Thus, nec-1 mitigated SAH-induced synaptic impairments in the hippocampus through the inhibition of necroptosis in connection with the CREB-BDNF pathway. This study may provide a new strategy for SAH patients in clinical practice.

## Introduction

Subarachnoid hemorrhage (SAH) is a devastating form of stroke that leads to a high mortality and disability rate (Bederson et al., 2009; Serrone et al., 2015). Nowadays, the study of SAH has focused on early brain injury (EBI), including the direct injury and the secondary pathophysiological changes of the brain within 72 h after the occurrence of SAH (Chen et al., 2015). There are multiple factors that lead to EBI, including cerebral ischemia, destruction of the blood brain barrier, cerebral edema, oxidative stress, inflammatory response, and apoptosis (Sehba et al., 2012). Thus, neuroprotective strategies against EBI is of the most significance for clinical treatment (Zhang X. et al., 2017).

Traditionally, it is believed that necrosis is an uncontrolled and accidental form of cell death (Fayaz et al., 2014). However, more recent evidence shows that necrosis can be well-controlled via a caspase-independent mechanism known as necroptosis (Hanson, 2016; Wang et al., 2017). This form of controlled cell death is activated when apoptosis is inhibited on account of the suppression of caspase activity, and it shares both the controlled mechanism seen in apoptosis and the morphological features seen in necrosis (Ou et al., 2017). Necroptosis plays a significant role in the pathophysiological process of many central nervous system diseases, including SAH (Zhang S. et al., 2017; Zille et al., 2017). The main goal of necroptosis is to reduce the number of abnormal cells present in brain tissue, which is important for the recovery of the brain from its injury after SAH. Previous studies have shown that necroptosis has a particular signal pathway, in which receptor-interacting proteins (RIP) 1&3 are involved (Festjens et al., 2007). RIP1 is identified as a significant regulator of necrotic death in cells where caspase was inhibited. It has been observed that RIP1 is an upstream kinase, and combined with RIP3 and induced by tumor necrosis factor (TNF)-α, this complex could regulate necroptosis (Galluzzi et al., 2009). Necrostatin-1(nec-1), a specific RIP1 inhibitor, could mitigate nerve injury by inhibiting necroptosis effectively (Chen et al., 2017; Zhou K. et al., 2017). Therefore, the neuroprotective functions of nec-1 as a result of suppressing necroptosis may provide new ideas for possible treatments for SAH (Zhang S. et al., 2017).

The hippocampus, which contributes mostly to learning and memory, suffers severe damage after SAH occurs (Shetty, 2014). Up to now, there are no studies focused on the process of necroptosis on neurons and synapses in the hippocampus after SAH. The morphological changes of the hippocampus after SAH and the mechanisms that lead to these changes are still unclear. Brain-derived neurotrophic factor (BDNF) and cAMP responsive element binding proteins (CREB) are reported to be involved in synaptic plasticity, which is believed to underlie memory processes (Seoane et al., 2011). Therefore, the links between nec-1 treatment and the CREB-BDNF pathway may provide a new therapeutic method to EBI after SAH.

## Materials and Methods

### Animals and the SAH Model

All the animal experimental procedures were approved by the Ethic Committee of Medical Department, Shandong University and Qilu Hospital. Necessary efforts were made to reduce the animals’ suffering. Male Wistar rats weighting 280–350 g were bought from the Laboratory Animal Center, Shandong University. The rats were housed under standard laboratory conditions for 1 week before treatment, with free access to food and water.

An experimental SAH model was made using a double blood injection model according to our previous study (Hu et al., 2016; Li et al., 2017b). Briefly, 3.5% isoflurane was used to induce anesthesia and 2.5% to maintain it. Two-hundred microliters of autologous blood, taken from the arteria cruralis, was injected into the cisterna magna within 3 min.

### Experimental Design and Drug Administration

A total of 159 rats were used in this study and 33 rats were used to detect RIP1&3. The other rats were randomly divided into four groups: the Sham group, the Sham+nec-1 group, the SAH group, the SAH+nec-1 group. One milligram of nec-1 was dissolved in 1 mL 1% dimethyl sulfoxide with 0.1 M phosphate buffer solution (PBS). After SAH occurred, 3 μL of nec-1 solution was injected intracerebroventricularly according to a previous report (Yin et al., 2015).

### Neurological Scores and Brain Water Content

Twenty-four hours postSAH, neurological scores were measured by the Garcia scoring system (Garcia et al., 1995; Sugawara et al., 2008). The following seven items were awarded different points: spontaneous activity (0–3 points), spontaneous movement of all limbs (0–3 points), movement of forelimbs (0–3 points), climbing the wall of the wire cage (1–3 points), reaction to touch on both sides of the trunk (1–3 points), and response to vibrissae touch (1–3 points). A double-blind measure was used.

The wet/dry method was used to determine the severity of brain edema. In sum, the brain was removed from the rat and weighed immediately. Then the brain was dried at 100°C for 48 h and weighed to determine the dry weight. [(Wet weight – Dry weight) / Wet weight] × 100% = Brain water content.

### Immunofluorescence Imaging

RIP1 and RIP3 were observed using immunofluorescence. The procedures are as follows: All the slides were blocked with 10% goat serum in PBS. Then, the slides were incubated overnight in a humidified chamber at 4°C with the following primary antibody (RIP1, 1:80, Abcam, Cambridge, MA, United States; and RIP3, 1:100, Abcam, MA, United States). After primary antibody incubation for one night, samples were washed 3 times by PBS and incubated with the appropriate fluorescent-conjugated secondary antibody (1:500 dilution, Sigma-Aldrich) for 1 h. Three microscope fields were chosen in which both RIP1&3, and DAPI were positive in the cells of the hippocampus. The number of active RIP1&3 and DAPI double positive cells was calculated as the mean of the numbers obtained from six pictures from each rat.

### Hematoxylin and Eosin (H&E) Staining

H&E staining was used to observe the morphological alterations after SAH. The paraffin-embedded sections were deparaffinized and rehydrated by immersing them for 5 min in xylene twice with agitation each time, and then immersing them in 100% ethanol, 95% ethanol, and 70% ethanol, successively. Thereafter, the slides were immersed for 30 s in water with manual agitation, and then dipped into a Coplin jar containing Mayer’s hematoxylin and agitated for 30 s. Next, the slide was rinsed in water for 1 min and stained with 1% eosin Y solution for 10–30 s with agitation. Finally, one or two drops of mounting medium were added and the slide was covered with a coverslip.

### Nissl Staining

Nissl staining was used to observe the morphological alterations as well. The main procedures used were as follows: First, the paraffin-embedded sections were deparaffinized and rehydrated as mentioned above. Then the slides were placed into 1:1 alcohol: chloroform overnight and dehydrated using 100 and 95% alcohol to distil the water. Next, the were stained slides in a 1% cresyl violet solution (warmed in a 37–50°C oven) for 5–10 min and then rinsed quickly in distilled water. The slides were differentiated in 95% ethyl alcohol for 20–30 min and checked microscopically.

### Silver Staining

Tissue were fixed in 4% paraformaldehyde for 30 min and rinsed twice in PBS for 5 min and once with distilled water. Then the slides were immersed in pre-warmed (37°C) 10% filtered silver nitrate and stained for 30 min. Six milliliters of concentrated ammonium hydroxide was added to the flask containing the silver nitrate solution, and the solution was cleared. Ammoniacal silver was poured onto the slides, which were stained for 15 min at 37°C, then exchanged for a 1% ammonium hydroxide solution for 3 min and then returning the ammoniacal silver to a flask and adding 25 drops of fresh developing solution. Next the slides were placed in this solution for 5 min and then transferred to 1% ammonium hydroxide solution for 3 min and to 5% sodium thiosulfate solution for 5 min. The slides were rinsed three times in distilled water, 5 min each. Finally, they were dehydrated and cleared with 95% ethyl alcohol, absolute alcohol, and xylene. The slides were mounted with a resinous medium and the best views were observed.

### Terminal Deoxynucleotidyl Transferase dUTP Nick End Labeling Staining

Neuron apoptosis in hippocampus was detected using Terminal deoxynucleotidyl transferase dUTP nick end labeling staining (TUNEL) according to the manufacturer’s protocol (KeyGEN BioTECH, china). Slides were then counter-stained with 4′,6-diamidino-2-phenylindole (DAPI). Three microscope fields of TUNEL-positive cells in hippocampus were chosen and imaged. Counting was performed in a blinded manner.

### Transmission Electron Microscopy

Fresh tissue blocks of the hippocampus are isolated preventing from physical damage. Put the tissue blocks in Fixative for TEM at 4°C for 2–4 h. Postfix with 1% OsO4 in 0.1 M PBS (pH 7.4) for 2 h at room temperature. Then dehydrate and infiltrate. Cut ultrathin sections (60–80 nm) with ultramicrotome. Stain sections with uranyl acetate in pure ethanol for 15 min, rinse with distilled water. Then stain with leas citrate for 15 min, rinse with distilled water. Allow sections air-dry overnight. Finally, the sections were examined under a Hitachi H-7700 TEM.

### Western Blot Analysis

Protein concentration in the hippocampus was determined as in our previous study. A quantity of 20–40 μg of protein was loaded onto an 8–15% gradient polyacrylamide gel, electrophoretically transferred to a polyvinylidene difluoride membrane and probed with the following primary antibodies: RIP1 antibody (1:1000, Abcam, United States); RIP3 antibody (1:1000, Abcam, United States); neuroligin (1:500, Abcam, United States); neurexin (1:500, Abcam, United States); PSD95 (1:1000, Cell Signaling); CREB (1:1000, Cell Signaling); phospho-CREB (p-CREB) (1:1000, Cell Signaling); BDNF (1:1000, Santa Cruz Biotechnology); β-actin (1:2000, Sigma-Aldrich) was used as an internal control. Secondary antibodies were horseradish peroxidase conjugated to mouse anti-rabbit/mouse immunoglobulin G (1:10,000, Sigma-Aldrich).

### Statistical Analysis

GraphPad prism 5 was used to analyze the data. All data are presented as the mean ± standard deviation. The immunofluorescence was analyzed by a one-way analysis of variance followed by Tukey’s *post hoc* analysis. The neurological scores were analyzed using a Kruskal–Wallis one-way analysis of variance on ranks followed by Dunn’s *post hoc* test; and a one-way analysis of variance followed by Tukey’s *post hoc* analysis was used for western blotting results. A *P* < 0.05 was considered a significant difference.

## Results

### Necroptosis Occurs in the Hippocampus After SAH

**FIGURE 1:**
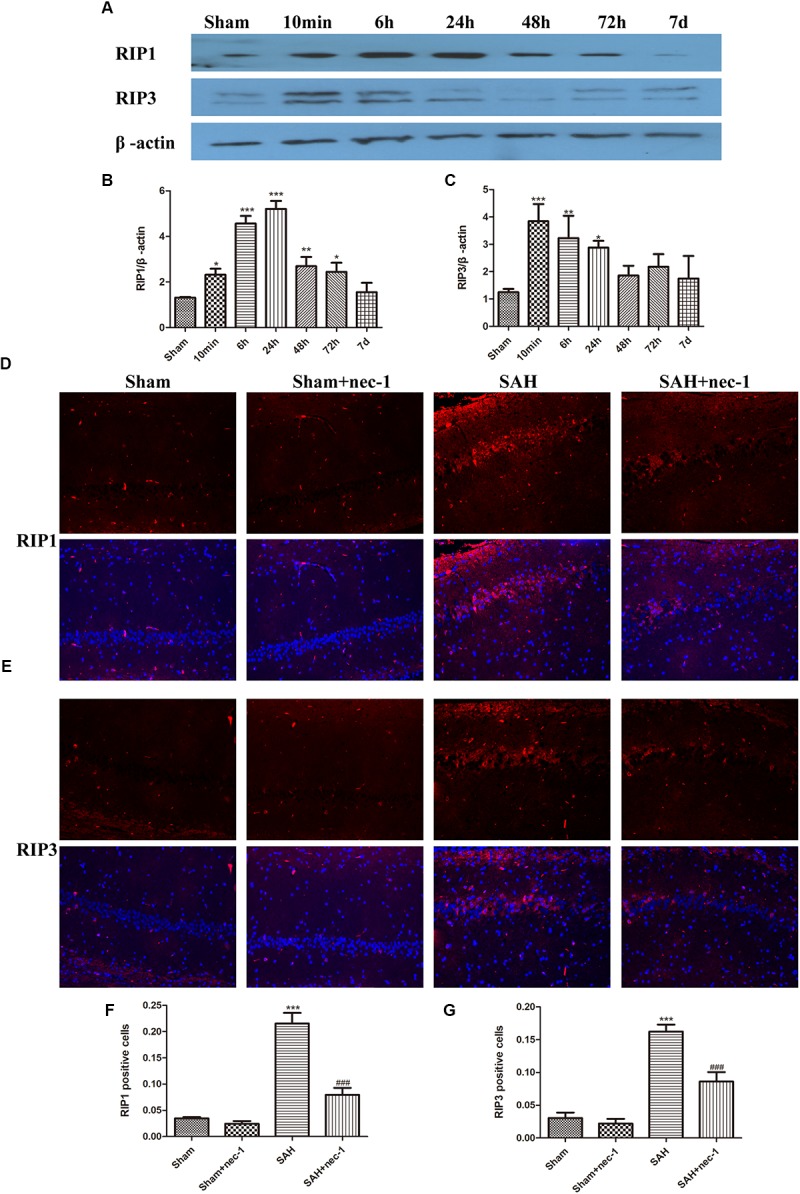
Necroptosis of hippocampal cells at different times post-SAH. (**A–C)** Western blotting results showed that the protein level of RIP1 increased 6 h after SAH and peaked at 24 h then decreased again. RIP3 also increased in 6 h after SAH and then decreased. Bar graphs showing the quantification of the protein levels of RIP1 and RIP3, and RIP1&3 were generated by Image-Pro Plus 6.0. The results are expressed as the RIP1&3/β-actin ratio (*n* = 3). The values represent the mean ± SD. ^∗^*P* < 0.05, ^∗∗^*P* < 0.01, ^∗∗∗^*P* < 0.001, SAH vs. Sham. **(D–G)** Immunofluorescence staining was used to detect RIP1 and RIP3. Three representative pictures were chosen in the CA1 area of hippocampus. Scale bar = 50 μm. Compared with the Sham group, RIP1 and RIP3 were significantly improved in the SAH group, and nec-1 treatment could reduce the expression of RIP1&3. The bar graphs showing the quantification of RIP1 or RIP3 positive cells (*n* = 4). Scale bar = 20 μm. ^∗∗∗^*P* < 0.001, SAH vs. Sham. ^###^*P* < 0.001, SAH+nec-1 vs. SAH.

Western blotting results showed that the protein level of RIP1 increased 6 h after SAH and peaked at 24 h and then decreased. RIP3 increased at 6 h after SAH and remained a high level until 24 h and then a decreasing trend was observed (Figures 1A–C). Immunofluorescence staining showed that there was little RIP1 and RIP3 expression in the Sham and Sham+nec-1 groups. In the SAH groups, these two proteins are highly expressed in CA1 (a subfield of the hippocampus proper) areas of the hippocampus and nec-1 reduced RIP1&3 expression significantly in the SAH+nec-1 group (Figures 1D–G). Additionally, we found that RIP1 and RIP3 are expressed in the CA3 subfield and the dentate gyrus of the hippocampus.

### Specific Necroptosis Inhibitor Nec-1 Improves Neurobehavior Scores and Attenuates Brain Edema

**FIGURE 2:**
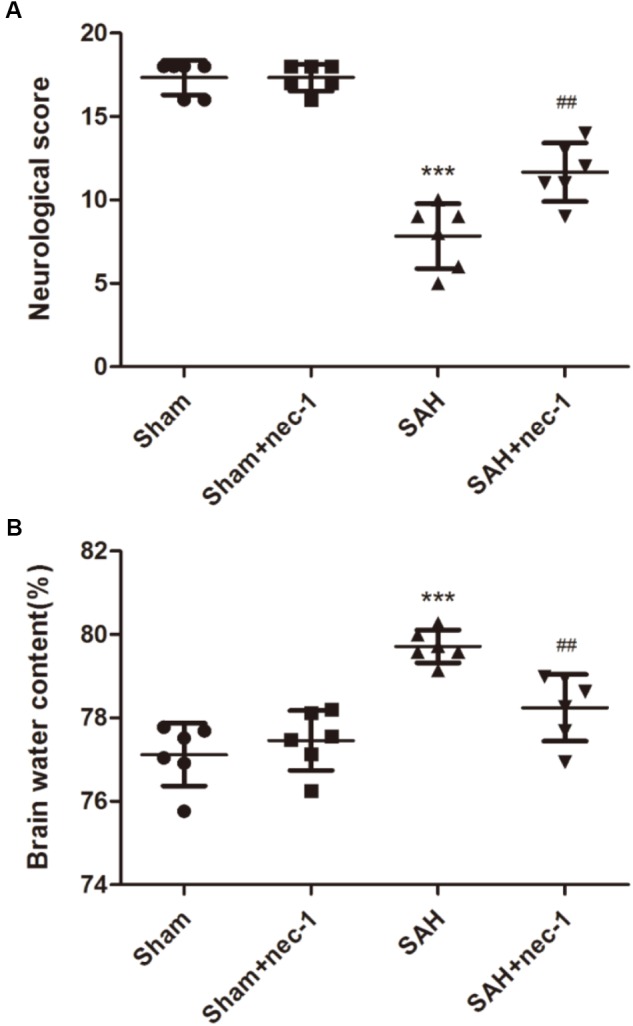
Specific necroptosis inhibitor improves neurobehavior scores and brain edema. **(A)** Compared with the Sham and Sham+nec-1 groups, the SAH group showed a lower neurobehavior score. In the Sham+nec-1 group, the injection of nec-1 significantly improved neurobehavior scores after SAH. ^∗∗∗^*P* < 0.001, Sham vs. SAH, ^##^*P* < 0.01, SAH+nec-1 vs. SAH. **(B)** Brain water content showed an obvious increase after SAH compared with the Sham group. However, nec-1 can effectively inhibit brain edema after SAH. ^∗∗∗^*P* < 0.001, Sham vs. SAH, ^##^*P* < 0.01, SAH+nec-1 vs. SAH.

Compared with the Sham group, the SAH group showed a lower neurobehavior score. In the SAH+nec-1 group, the injection of nec-1 significantly improved neurobehavior scores after SAH (Figure 2A). In addition, brain water content showed an obvious increase in the SAH group compared with the Sham group. However, nec-1 can effectively alleviate brain edema after SAH (Figure 2B).

### Nec-1 Improves the Pathological Changes of the Hippocampus After SAH

**FIGURE 3:**
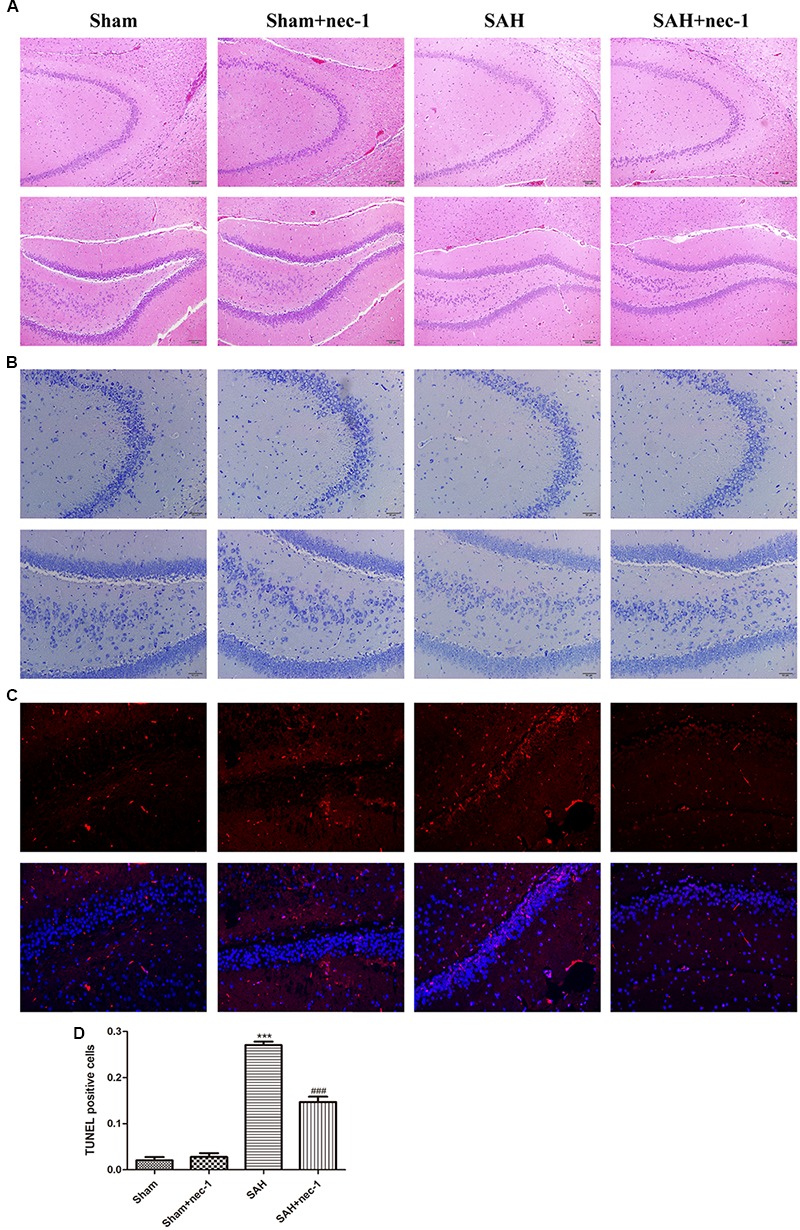
Nec-1 improves the pathological changes of the hippocampus after SAH. **(A)** In the H&E slices, the death of neurons and the damage to the hippocampus was clearly observed after SAH, both in amount of neurons and by the morphological structure of the hippocampus. Scale bar = 100 μm. **(B)** In the Nissl slices, the death of neurons and the damage of hippocampus was also clearly observed; the structure of the hippocampus was destroyed both in the CA3 and DG areas. Scale bar = 50 μm. **(C,D)** In TUNEL staining, the SAH group showed more TUNEL-positive cells than the Sham group. In the Sham+nec-1 group, the number of TUNEL-positive cells decreased compared to the SAH group. ^∗∗∗^*P* < 0.001, Sham vs. SAH, ^###^*P* < 0.001, SAH+nec-1 vs. SAH.

**FIGURE 4:**
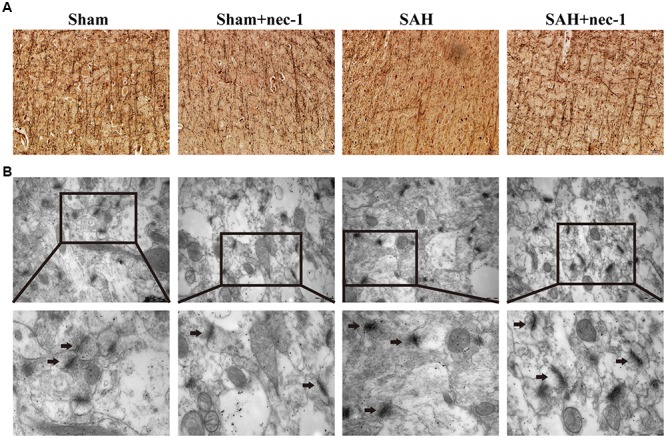
Nec-1 rescues synaptic impairments after SAH. **(A)** In silver staining, the impairments after SAH were clearly observed, and the number of nerve fibers in the SAH group decreased significantly. Nec-1 could increase the number of nerve fibers. Scale bar = 50 μm. **(B)** Morphology of the synapse was observed by electron microscope. In the Sham and Sham+nec-1 groups, presynaptic structures were clearly seen. However, in the SAH group, the structure of the synapse was damaged. The injection of nec-1 could effectively attenuate the damage. The arrows showed the structure of the presynaptic and postsynaptic membranes, and the synaptic cleft. Scale bar = 500 nm.

In the CA3 and dentate gyrus areas of the hippocampus, the amount of neuronal death and the damage to the morphological changes of the hippocampus were clearly observed after SAH by H&E staining. In the SAH+nec-1 group, nec-1 treatment showed an obvious reverse on the damage caused by SAH (Figure 3A). In the SAH group, Nissl staining also showed severe damage in the CA3 and dentate gyrus areas of the hippocampus. In the SAH+nec-1 group, we observed an increase in the number of neurons, as well as evident improvement in the structure of the hippocampus, compared to the SAH group (Figure 3B). The same results are seen in the CA1 area of the hippocampus. Terminal deoxynucleotidyl transferase dUTP nick end labeling showed that few apoptotic cells were observed in the Sham and Sham+nec-1 groups, whereas there was a large number of apoptotic cells in the SAH group, implying that nec-1 treatment could effectively reduce apoptosis (Figures 3C,D). Silver staining was used to observe the nerve fibers, which markedly decreased after SAH compared to the Sham group. After nec-1 treatment, the amount of nerve fibers increased, similar to the amount seen in the Sham group (Figure 4A).

### Nec-1 Attenuate Synaptic Impairments

Morphological changes of the synapse were detected using an electron microscope. In the Sham and Sham+nec-1 groups, the presynaptic membrane, postsynaptic membrane, and synaptic cleft were clearly seen. There were visible synaptic vesicles in the presynaptic membrane with neurotransmitters in them, and chondriosomes were observed clearly. However, in the SAH group, the structures of the synapses were damaged, indicating that there is injury to synapses after SAH. Additionally, we observed disruption of the presynaptic and postsynaptic membranes, and the synaptic cleft was almost not visible in the SAH group. However, after nec-1 treatment, the structure of synapses improved and became more similar to those seen in the Sham group. In the SAH+nec-1 group, the synaptic membrane and cleft were clearly seen compared to the SAH group (Figure 4B).

### Nec-1 Protects Functional Proteins of the Hippocampus

**FIGURE 5:**
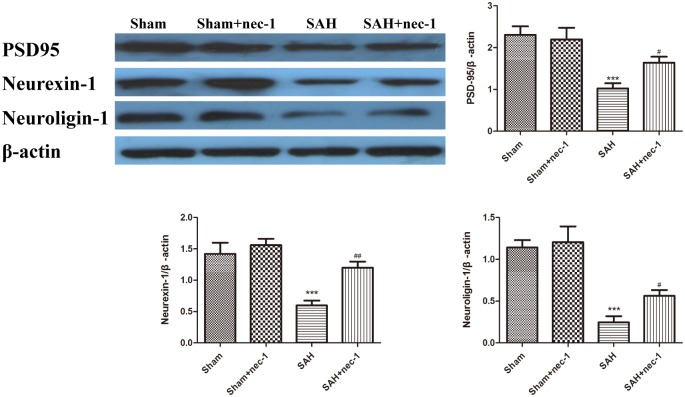
The effect of nec-1 on synaptic protein expression. The protective impact of Nec-1 on synapses was detected by western blotting. The results revealed a decrease of synaptic proteins after SAH occurred compared to the Sham group. Treatment with Nec-1 increased the expression of synaptic proteins. Bar graphs showing the quantification of the protein levels of PSD-95, neurexin, and neuroligin. The results are expressed as the PSD95, neurexin-1 and neuroligin-1/β-actin ratio (*n* = 3). The values represent the mean ± SD. ^∗∗∗^*P* < 0.001 SAH vs. Sham, ^#^*P* < 0.05, ^##^*P* < 0.01, SAH+nec-1 vs. SAH.

The protective impact of nec-1 on the hippocampus was detected using western blotting. The results revealed an obvious decrease of the synaptic proteins neuroligin, neurexin, and PSD95, after SAH occurred, compared to the Sham group. Treatment with nec-1 increased the expression of these synaptic proteins. In other words, injection of nec-1 can reverse the synaptic damage caused by SAH (Figure 5).

### Nec-1 Has an Influence on the CREB-BDNF Pathway

**FIGURE 6:**
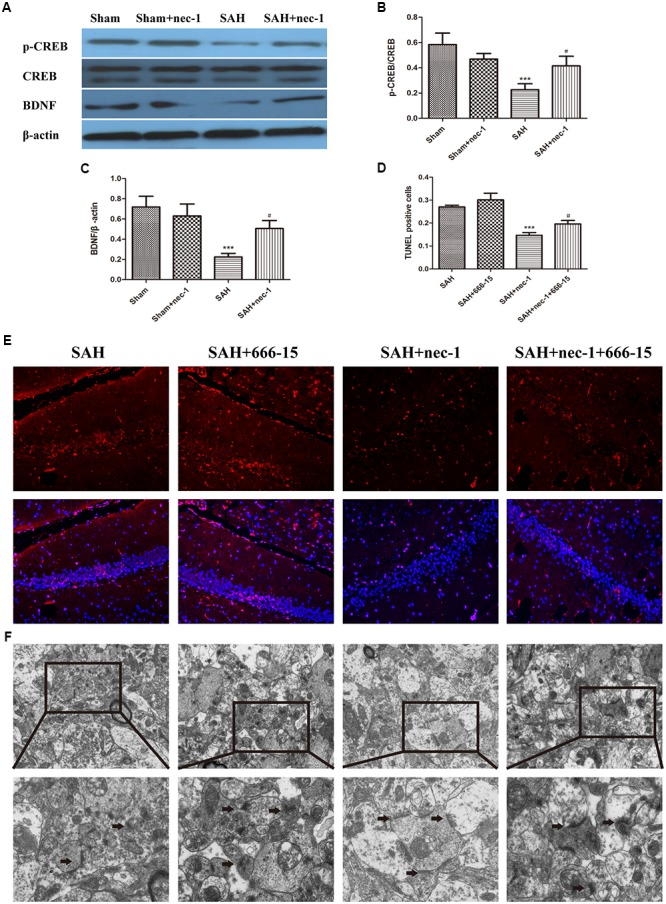
Pathways that nec-1 relies on to exert neuroprotective effects. **(A–C)** Western blotting was used to detect CREB, p-CREB, and BDNF. After SAH occurred, these three proteins showed an obvious decrease. However, the injection of Nec-1 increased expression of these three proteins. Bar graphs showing the quantification of the protein levels of p-CREB, CREB, and BDNF. The results are expressed as the p-CREB/CREB ratio and BDNF/β-actin ratio (*n* = 3). The values represent the mean ± SD., ^∗∗∗^*P* < 0.001, SAH vs. Sham, ^#^*P* < 0.05 SAH+nec-1 vs. SAH. **(D,E)** TUNEL showed the apoptotic cells. In the SAH+nec-1 group, there are few TUNEL-positive cells, whereas there are many in the SAH+nec-1+666-15 group. Scale bar = 20 μm. **(F)** Transmission electron microscopy showed the structure of synapses. In the SAH and SAH+nec-1 groups, presynaptic structures were damaged and nec-1 treatment could alleviate synaptic impairment. In the SAH+nec-1+666-15 group, the protective effect of nec-1 was reversed. The arrows showed the structure of the presynaptic and postsynaptic membranes, and the synaptic cleft. Scale bar = 500 nm.

Western blotting was used to detect CREB, p-CREB, and BDNF, which are crucial proteins in the CREB-BDNF pathway that may play a neuroprotective role in reducing the synaptic impairments after SAH. After SAH occurred, BDNF and p-CREB markedly decreased, however, an injection of nec-1 could upregulate p-CREB and BDNF expression in the SAH+nec-1 group, compared with the SAH group (Figures 6A–C). In addition, compared to SAH+nec-1 group, the injection of 666-15 could increase the terminal deoxynucleotidyl transferase dUTP nick end labeling-positive cells in the hippocampus in the SAH+nec-1+666-15 group (Figures 6D,E). In the SAH+nec-1+666-15 group, the structure of synapses deteriorated compared to SAH+nec-1 group, with poorly visible synaptic membranes and cleft (Figure 6F).

## Discussion

In this study, we report that SAH induced necroptosis because of a subsequent increase in the levels of RIP1 and RIP3, however, inhibition of necroptosis by nec-1 ameliorated the morphological damage to the hippocampus. In addition, the number of synaptic-associated proteins were reduced after SAH but obviously increased again after an injection of nec-1. Furthermore, p-CREB and BDNF levels were enhanced, and a specific CREB inhibitor, i.e., 666-15, could counteract the effect of nec-1, indicating that CREB-BDNF could be one potential pathway influenced by nec-1 treatment.

It has been demonstrated that EBI is the most devastating pathophysiological process after an aneurysmal SAH, instead of cerebral vasospasm (Fujii et al., 2013). EBI within 72 h after SAH occurs via complex mechanisms, including cerebral ischemia, destruction of blood-brain barrier, cerebral edema, oxidative stress, inflammatory response, and apoptosis (Van Lieshout et al., 2017). Although EBI is the most important process leading to unfavorable outcomes after SAH, the underlying pathophysiology has not been well understood. Cell death in EBI can occur via several ways, such as necrosis, apoptosis, and autophagy (Ji and Chen, 2016). Necroptosis, a kind of programmed cell death, was shown to play an important role in SAH-induced neuron death (Zille et al., 2017). Some studies have found that the inhibition of necroptosis via nec-1 could attenuate EBI after SAH in rats (Shen et al., 2017). The inhibition of necroptosis may shed new light on SAH treatment (Kooijman et al., 2014). However, the neuroprotective effect of nec-1 on the hippocampus has not been well clarified. In this study, after SAH occurred, necroptosis was activated in the hippocampus following the increase of RIP1 and RIP3, sharing similar results with other studies (Watson et al., 2014). RIP1 and RIP3 are the upstream proteins in necroptosis, a kind of TNF-induced programmed necrosis (Zhou K. et al., 2017). After being activated, RIP3 is phosphorylated by activated RIP1 and combines with RIP1 to form an amyloid-like necrosome complex (Li et al., 2012). The complex is stabilized by the phosphorylation of RIP1 and RIP3, and then the necrosome complex triggers a series of downstream pathways (Zhou Z. et al., 2017). In our study, after the injection of nec-1, brain edema was decreased, and neurological scores increased significantly, suggesting that the inhibition of necroptosis could attenuate the damage caused by EBI after SAH.

The hippocampus is an important area of brain involved in the functions of learning, memory, and mood. It is also one of the regions of the brain which has a robust plasticity after injury (Zhou H. et al., 2012). Synapses in the hippocampus are the fundamental structure of learning and memory (Shapiro and Eichenbaum, 1999). Synapses are formed by the tight attachment of presynaptic and postsynaptic specializations, which permit a neuron to pass an electrical or chemical signal to another neuron (Han et al., 2014). Multiple factors can result in hippocampal injury, including SAH. In this study, we found that there was obvious injury to the hippocampus after SAH, i.e., the number of neurons decreased, and the structure of the hippocampus was damaged. However, we also found that nec-1 treatment could effectively attenuate the resulting hippocampal structural damage. Synapses in the hippocampus also suffered severe damage after SAH. However, the structure of synapses showed significant improvement after the injection of nec-1. The amelioration of synaptic injury, by inhibiting necroptosis in the hippocampus, could be a new treatment for cognitive disorders after SAH.

Previous studies have shown that the expression of neurexin-1 and neuroligin-1, and the interaction between them, are closely related to cognitive function after injury (Shen et al., 2015). Neurexins are controlled by diverse trans-synaptic signaling molecules and have functions to determine the properties of synapses (Sudhof, 2017). Presynaptic neurexins regulate synapse properties via differential binding to multifarious postsynaptic ligands, including neuroligins which are essential for postsynaptic specialization and synaptic function (Constance et al., 2018). Postsynaptic density 95 is one of the most abundant components of the synapse and functions as a scaffolding protein to regulate synaptic plasticity and stabilize synaptic structure (Elias et al., 2008). In the present study, we found that after SAH occurred, neurexin-1, neuroligin-1, and postsynaptic density 95 were decreased, indicating that the synapses suffered severe damage. However, treatment with nec-1 increased these three synaptic proteins, which means that nec-1 will mitigate the impairment of synapses that occurs after SAH.

It has been reported that BDNF and CREB are closely involved in the impairment seen with brain injury after SAH (Li et al., 2017a,b). BDNF is a neuroprotective protein involved in synaptic plasticity and is known to be the basis of memory processes (Seoane et al., 2011). One pathway in which BDNF plays a role in improving the plasticity of synapses might be in the activation of the transcription factor CREB (Kim et al., 2018). Phosphorylation of CREB was reported to be necessary for plasticity and recognition memory mechanisms (Liraz-Zaltsman et al., 2018). Exposure of neurons to BDNF can lead to activation of CREB, and the activation of CREB transcriptional processes has been linked to synaptic plasticity, learning and memory (Saura and Cardinaux, 2017). Until now, no investigations have focused on the effects of nec-1 on CREB and BDNF. In the present study, we observed obvious downregulation of p-CREB and BDNF after SAH, and then an increase again after nec-1 injection. 666-15 is reported to be a small molecule inhibitor of CREB-mediated gene transcription (Xie et al., 2017). Pharmacological inhibition of CREB by 666-15 is well-tolerated *in vivo* (Li et al., 2016). In the present study, treatment with 666-15 could reverse the neuroprotective effect of nec-1 according to terminal deoxynucleotidyl transferase dUTP nick end labeling and transmission electron microscopy, which suggests that the inhibition of necroptosis may have a potential interrelationship with the CREB-BDNF pathway.

However, there are still some limitations to our study. Firstly, apoptosis and autophagy share similar signal pathways with necroptosis, and the mechanism of nec-1 on regulating apoptosis and autophagy has not been studied. Secondly, EBI after SAH is a complex process that multiple factors may affect that we have not explored. Furthermore, the concrete mechanism of how nec-1 affects the CREB-BDNF pathway is still unclear. These would be the focus of our future research.

In summary, nec-1 treatment may alleviate EBI development induced by experimental SAH in rats by inhibiting necroptosis. In the meantime, improvements to the structure of the hippocampus were observed after initial damage from SAH, and the damage of synapses was alleviated with the upregulation of CREB–BDNF expression. Therefore, the inhibition of necroptosis may provide a new therapeutic method to EBI.

## Ethics Statement

This study was carried out in accordance with the recommendations of International Guiding Principles for Animal Research, as stipulated by the World Health Organization (1985). The protocol was approved by the Ethic Committee of Medical Department, Shandong University and Qilu Hospital.

## Author Contributions

GL and ZW were involved in study design, data interpretation, writing and revising of the manuscript. CY and TL performed the majority of the laboratory work and analyzed the data. HX and LD contributed to behavioral testing. LW and YX contributed to animal model. XB, DX, HY, and JQ contributed to Western blot. All authors approved the final version of the manuscript.

## Conflict of Interest Statement

The authors declare that the research was conducted in the absence of any commercial or financial relationships that could be construed as a potential conflict of interest.

## Abbreviations

EBI: early brain injury
Nec-1: Necrostatin-1
RIP1: receptor-interacting protein 1
RIP3: receptor-interacting protein 3

## References

[B1] Bederson J. B. Connolly E. S. Jr. Batjer H. H. Dacey R. G. Dion J. E. Diringer M. N. (2009). Guidelines for the management of aneurysmal subarachnoid hemorrhage: a statement for healthcare professionals from a special writing group of the stroke council, american heart association. *Stroke* 40 994–1025. 10.1161/STROKEAHA.108.191395 19164800

[B2] Chen F. Su X. Lin Z. Lin Y. Yu L. Cai J. (2017). Necrostatin-1 attenuates early brain injury after subarachnoid hemorrhage in rats by inhibiting necroptosis. *Neuropsychiatr. Dis. Treat.* 13 1771–1782. 10.2147/NDT.S140801 28744127 PMC5511017

[B3] Chen S. Wu H. Tang J. Zhang J. Zhang J. H. (2015). Neurovascular events after subarachnoid hemorrhage: focusing on subcellular organelles. *Acta Neurochir. Suppl.* 120 39–46. 10.1007/978-3-319-04981-6_7 25366597 PMC4373344

[B4] Constance W. D. Mukherjee A. Fisher Y. E. Pop S. (2018). Neurexin and neuroligin-based adhesion complexes drive axonal arborisation growth independent of synaptic activity. *eLife* 7:e31659. 10.7554/eLife.31659 29504935 PMC5869020

[B5] Elias G. M. Elias L. A. Apostolides P. F. Kriegstein A. R. Nicoll R. A. (2008). Differential trafficking of AMPA and NMDA receptors by SAP102 and PSD-95 underlies synapse development. *Proc. Natl. Acad. Sci. U.S.A.* 105 20953–20958. 10.1073/pnas.0811025106 19104036 PMC2634944

[B6] Fayaz S. M. Suvanish Kumar V. S. Rajanikant G. K. (2014). Necroptosis: who knew there were so many interesting ways to die? *CNS Neurol. Disord. Drug Targets* 13 42–51. 10.2174/18715273113126660189 24152329

[B7] Festjens N. Vanden Berghe T. Cornelis S. Vandenabeele P. (2007). RIP1, a kinase on the crossroads of a cell’s decision to live or die. *Cell Death Differ.* 14 400–410. 10.1038/sj.cdd.4402085 17301840

[B8] Fujii M. Yan J. Rolland W. B. Soejima Y. Caner B. Zhang J. H. (2013). Early brain injury, an evolving frontier in subarachnoid hemorrhage research. *Transl. Stroke Res.* 4 432–446. 10.1007/s12975-013-0257-2 23894255 PMC3719879

[B9] Galluzzi L. Kepp O. Kroemer G. (2009). RIP kinases initiate programmed necrosis. *J. Mol. Cell Biol.* 1 8–10. 10.1093/jmcb/mjp007 19679643

[B10] Garcia J. H. Wagner S. Liu K. F. Hu X. J. (1995). Neurological deficit and extent of neuronal necrosis attributable to middle cerebral artery occlusion in rats: statistical validation. *Stroke* 26 627–635. 10.1161/01.STR.26.4.627 7709410

[B11] Han S. M. Wan H. Kudo G. Foltz W. D. Vines D. C. Green D. E. (2014). Molecular alterations in the hippocampus after experimental subarachnoid hemorrhage. *J. Cereb. Blood Flow Metab.* 34 108–117. 10.1038/jcbfm.2013.170 24064494 PMC3887350

[B12] Hanson B. (2016). Necroptosis: a new way of dying? *Cancer Biol. Ther.* 17 899–910. 10.1080/15384047.2016.1210732 27434654 PMC5036404

[B13] Hu Q. Li T. Wang L. Xie Y. Liu S. Bai X. (2016). Neuroprotective effects of a smoothened receptor agonist against early brain injury after experimental subarachnoid hemorrhage in rats. *Front. Cell. Neurosci.* 10:306. 10.3389/fncel.2016.00306 28149272 PMC5241312

[B14] Ji C. Chen G. (2016). Signaling pathway in early brain injury after subarachnoid hemorrhage: news update. *Acta Neurochir. Suppl.* 121 123–126. 10.1007/978-3-319-18497-5_21 26463934

[B15] Kim Y. R. Kwon M. Y. Pak M. E. Park S. H. Baek J. U. Choi B. T. (2018). Beneficial effects of gagam-palmultang on scopolamine-induced memory deficits in mice. *Evid. Based Complement. Alternat. Med.* 2018:3479083. 10.1155/2018/3479083 29670659 PMC5835292

[B16] Kooijman E. Nijboer C. H. Van Velthoven C. T. Kavelaars A. Kesecioglu J. Heijnen C. J. (2014). The rodent endovascular puncture model of subarachnoid hemorrhage: mechanisms of brain damage and therapeutic strategies. *J. Neuroinflammation* 11:2. 10.1186/1742-2094-11-2 24386932 PMC3892045

[B17] Li B. X. Gardner R. Xue C. Qian D. Z. Xie F. Thomas G. (2016). Systemic inhibition of CREB is well-tolerated in vivo. *Sci. Rep.* 6:34513. 10.1038/srep34513 27694829 PMC5046085

[B18] Li J. Mcquade T. Siemer A. B. Napetschnig J. Moriwaki K. Hsiao Y. S. (2012). The RIP1/RIP3 necrosome forms a functional amyloid signaling complex required for programmed necrosis. *Cell* 150 339–350. 10.1016/j.cell.2012.06.019 22817896 PMC3664196

[B19] Li T. Liu H. Xue H. Zhang J. Han X. Yan S. (2017a). Neuroprotective effects of hydrogen sulfide against early brain injury and secondary cognitive deficits following subarachnoid hemorrhage. *Brain Pathol.* 27 51–63. 10.1111/bpa.12361 26822402 PMC8029220

[B20] Li T. Wang L. Hu Q. Liu S. Bai X. Xie Y. (2017b). Neuroprotective roles of l-Cysteine in attenuating early brain injury and improving synaptic density via the CBS/H2S pathway following subarachnoid hemorrhage in rats. *Front. Neurol.* 8:176. 10.3389/fneur.2017.00176 28512446 PMC5411453

[B21] Liraz-Zaltsman S. Slusher B. Atrakchi-Baranes D. Rosenblatt K. Friedman Levi Y. Kesner E. (2018). Enhancement of brain d-Serine mediates recovery of cognitive function after traumatic brain injury. *J. Neurotrauma* 35 1667–1680. 10.1089/neu.2017.5561 29648983

[B22] Ou L. Lin S. Song B. Liu J. Lai R. Shao L. (2017). The mechanisms of graphene-based materials-induced programmed cell death: a review of apoptosis, autophagy, and programmed necrosis. *Int. J. Nanomedicine* 12 6633–6646. 10.2147/IJN.S140526 28924347 PMC5595361

[B23] Saura C. A. Cardinaux J. R. (2017). Emerging roles of CREB-regulated transcription coactivators in brain physiology and pathology. *Trends Neurosci.* 40 720–733. 10.1016/j.tins.2017.10.002 29097017

[B24] Sehba F. A. Hou J. Pluta R. M. Zhang J. H. (2012). The importance of early brain injury after subarachnoid hemorrhage. *Prog. Neurobiol.* 97 14–37. 10.1016/j.pneurobio.2012.02.003 22414893 PMC3327829

[B25] Seoane A. Tinsley C. J. Brown M. W. (2011). Interfering with perirhinal brain-derived neurotrophic factor expression impairs recognition memory in rats. *Hippocampus* 21 121–126. 10.1002/hipo.20763 20087891 PMC4258639

[B26] Serrone J. C. Maekawa H. Tjahjadi M. Hernesniemi J. (2015). Aneurysmal subarachnoid hemorrhage: pathobiology, current treatment and future directions. *Expert Rev. Neurother.* 15 367–380. 10.1586/14737175.2015.1018892 25719927

[B27] Shapiro M. L. Eichenbaum H. (1999). Hippocampus as a memory map: synaptic plasticity and memory encoding by hippocampal neurons. *Hippocampus* 9 365–384. 10.1002/(SICI)1098-1063(1999)9:4<365::AID-HIPO4>3.0.CO;2-T 10495019

[B28] Shen H. Chen Z. Wang Y. Gao A. Li H. Cui Y. (2015). Role of neurexin-1beta and neuroligin-1 in cognitive dysfunction after subarachnoid hemorrhage in rats. *Stroke* 46 2607–2615. 10.1161/STROKEAHA.115.009729 26219651 PMC4542569

[B29] Shen H. Liu C. Zhang D. Yao X. Zhang K. Li H. (2017). Role for RIP1 in mediating necroptosis in experimental intracerebral hemorrhage model both in vivo and in vitro. *Cell Death Dis.* 8:e2641. 10.1038/cddis.2017.58 28252651 PMC5386555

[B30] Shetty A. K. (2014). Hippocampal injury-induced cognitive and mood dysfunction, altered neurogenesis, and epilepsy: can early neural stem cell grafting intervention provide protection? *Epilepsy Behav.* 38 117–124. 10.1016/j.yebeh.2013.12.001 24433836 PMC4742318

[B31] Sudhof T. C. (2017). Synaptic neurexin complexes: a molecular code for the logic of neural circuits. *Cell* 171 745–769. 10.1016/j.cell.2017.10.024 29100073 PMC5694349

[B32] Sugawara T. Ayer R. Jadhav V. Zhang J. H. (2008). A new grading system evaluating bleeding scale in filament perforation subarachnoid hemorrhage rat model. *J. Neurosci. Methods* 167 327–334. 10.1016/j.jneumeth.2007.08.004 17870179 PMC2259391

[B33] Van Lieshout J. H. Dibue-Adjei M. Cornelius J. F. Slotty P. J. Schneider T. Restin T. (2017). An introduction to the pathophysiology of aneurysmal subarachnoid hemorrhage. *Neurosurg. Rev.* 41 917–930. 10.1007/s10143-017-0827-y 28215029

[B34] Wang T. Jin Y. Yang W. Zhang L. Jin X. Liu X. (2017). Necroptosis in cancer: an angel or a demon? *Tumour Biol.* 39:1010428317711539. 10.1177/1010428317711539 28651499

[B35] Watson W. H. Burke T. J. Doll M. A. Mcclain C. J. (2014). S-adenosylhomocysteine inhibits NF-kappaB-mediated gene expression in hepatocytes and confers sensitivity to TNF cytotoxicity. *Alcohol. Clin. Exp. Res.* 38 889–896. 10.1111/acer.12315 24224954 PMC4035050

[B36] Xie F. Li B. X. Xiao X. (2017). Design, synthesis and biological evaluation of regioisomers of 666-15 as inhibitors of CREB-mediated gene transcription. *Bioorg. Med. Chem. Lett.* 27 994–998. 10.1016/j.bmcl.2016.12.078 28073675 PMC5296214

[B37] Yin B. Xu Y. Wei R. L. He F. Luo B. Y. Wang J. Y. (2015). Inhibition of receptor-interacting protein 3 upregulation and nuclear translocation involved in Necrostatin-1 protection against hippocampal neuronal programmed necrosis induced by ischemia/reperfusion injury. *Brain Res.* 1609 63–71. 10.1016/j.brainres.2015.03.024 25801119

[B38] Zhang S. Tang M. B. Luo H. Y. Shi C. H. Xu Y. M. (2017). Necroptosis in neurodegenerative diseases: a potential therapeutic target. *Cell Death Dis.* 8:e2905. 10.1038/cddis.2017.286 28661482 PMC5520937

[B39] Zhang X. Wu Q. Zhang Q. Lu Y. Liu J. Li W. (2017). Resveratrol attenuates early brain injury after experimental subarachnoid hemorrhage via inhibition of NLRP3 inflammasome activation. *Front. Neurosci.* 11:611. 10.3389/fnins.2017.00611 29163015 PMC5675880

[B40] Zhou H. Chen L. Gao X. Luo B. Chen J. (2012). Moderate traumatic brain injury triggers rapid necrotic death of immature neurons in the hippocampus. *J. Neuropathol. Exp. Neurol.* 71 348–359. 10.1097/NEN.0b013e31824ea078 22437344 PMC3311037

[B41] Zhou K. Shi L. Wang Z. Zhou J. Manaenko A. Reis C. (2017). RIP1-RIP3-DRP1 pathway regulates NLRP3 inflammasome activation following subarachnoid hemorrhage. *Exp. Neurol.* 295 116–124. 10.1016/j.expneurol.2017.06.003 28579326

[B42] Zhou Z. Lu B. Wang C. Wang Z. Luo T. Piao M. (2017). RIP1 and RIP3 contribute to shikonin-induced DNA double-strand breaks in glioma cells via increase of intracellular reactive oxygen species. *Cancer Lett.* 390 77–90. 10.1016/j.canlet.2017.01.004 28108311

[B43] Zille M. Karuppagounder S. S. Chen Y. Gough P. J. Bertin J. Finger J. (2017). Neuronal death after hemorrhagic stroke in vitro and in vivo shares features of ferroptosis and necroptosis. *Stroke* 48 1033–1043. 10.1161/STROKEAHA.116.015609 28250197 PMC5613764

